# Imaging the Ultrafast Photoelectron Transfer Process in Alizarin-TiO_2_

**DOI:** 10.3390/molecules200813830

**Published:** 2015-07-30

**Authors:** Tatiana Gomez, Gunter Hermann, Ximena Zarate, Jhon Fredy Pérez-Torres, Jean Christophe Tremblay

**Affiliations:** 1 Dirección de Postgrado e Investigación, Universidad Autónoma de Chile, Llano Subercaceaux 2801, San Miguel, Santiago, Chile; E-Mail: ximena.zarate@uautonoma.cl; 2 Institut für Chemie und Biochemie, Freie Universität Berlin, Takustraße 3, 14195 Berlin, Germany; E-Mails: gunter.hermann@fu-berlin.de (G.H.); jperez@zedat.fu-berlin.de (J.F.P.-T.)

**Keywords:** dye-sensitized solar cell, optical spectra, electronic flux density

## Abstract

In this work, we adopt a quantum mechanical approach based on time-dependent density functional theory (TDDFT) to study the optical and electronic properties of alizarin supported on TiO${}_{2}$ nano-crystallites, as a prototypical dye-sensitized solar cell. To ensure proper alignment of the donor (alizarin) and acceptor (TiO${}_{2}$ nano-crystallite) levels, static optical excitation spectra are simulated using time-dependent density functional theory in response. The ultrafast photoelectron transfer from the dye to the cluster is simulated using an explicitly time-dependent, one-electron TDDFT ansatz. The model considers the δ-pulse excitation of a single active electron localized in the dye to the complete set of energetically accessible, delocalized molecular orbitals of the dye/nano-crystallite complex. A set of quantum mechanical tools derived from the transition electronic flux density is introduced to visualize and analyze the process in real time. The evolution of the created wave packet subject to absorbing boundary conditions at the borders of the cluster reveal that, while the electrons of the aromatic rings of alizarin are heavily involved in an ultrafast charge redistribution between the carbonyl groups of the dye molecule, they do not contribute positively to the electron injection and, overall, they delay the process.

## 1. Introduction

Converting the endless supply of freely available solar energy into electric power appears to be one of the most economically viable strategies to generate current from alternative energy sources in the long term, although concerns were raised about the efficiency of the process. In this context, Dye Sensitized Solar Cells (DSSC) have attracted significant interest since they can directly convert sunlight into electrical energy at low cost and with comparatively high efficiency [1,2,3,4,5]. DSSC were pioneered by Bryan O’Regan and Michael Grätzel in the late 1980s, and the first high efficiency DSSC were introduced in 1991 [2]. Modern DSSC are generally based on nanostructured mesoporous metal oxide films or nanoparticles, usually titanium dioxide (TiO${}_{2}$), sensitized to harvest the visible light by an adsorbed dye molecule. The working mechanism of DSSC is usually understood to undergo various processes that can be divided in five distinct steps [6,7] (1) the photoexcitation of the dye upon which it harvests the energy from the sun; (2) the injection of light-excited electrons into the conduction band of the metal oxide; (3) the migration of the electrons in the TiO${}_{2}$ toward the anode; (4) the regeneration of the oxidized dye by a redox pair in the surrounding electrolyte; and (5) the regeneration of the redox pair by its reduction at the cathode. The second part of this process is a key stage that allows the efficient harvesting of solar energy, as the injection of light-excited electrons from the dye into the semiconductor conduction band should lead to a very fast charge separation for the creation of a potential bias. Experimentally, the injection rate has been found to depend on the electronic properties of both the dye and the semiconductor, as well as the distance between them [8], and the efficiency of the solar cells thus depends strongly on the optical and electronic properties of the employed dye. Among the wide variety of dyes that have been investigated since the early days of the DSSC, one the best conversion efficiencies has been achieved using ruthenium-based dyes in combination with the iodide/triiodide redox couple [9,10]. The main disadvantages of these molecules are that ruthenium is a rare, expensive, and toxic metal, and that molecular modification of these dyes is an arduous task due to their complicated synthesis routes.

In recent years, alternatives to ruthenium sensitizers have emerged in the form of organic dyes, which can achieve similar photon-to-current conversion efficiencies while remaining inexpensive and easy to synthesize [4,8,11,12,13]. As such, alizarin has provided a fertile playground for experimentalists and theoreticians altogether. Numerous experimental studies have yielded a wealth of information about its optical properties in various environments: as a colloidal form in solution [14,15,16,17], and adsorbed on thin films [18] and at nanoparticles [19]. These findings were also investigated theoretically, both from the spectroscopic and dynamical perspectives [4,8,12,19]. An important aspect for understanding the DSSC mechanism is that of the structure at the dye-metal oxide interface and its influence on the optical adsorption spectrum of the dye. In particular, a large red shift in the absorption bands of alizarin is observed upon adsorption on the dye, which is of critical importance for the light-harvesting properties of the complex requiring proper band alignment between the dye molecule and the TiO${}_{2}$ nano-crystallite. In early investigations, Prezhdo and co-workers [20,21,22] have established the suitability of time-dependent density functional theory for reproducing the optical spectra of alizarin and catechol on titanium oxide, as modeled using a single titanium atom. For colloidal systems, Sánchez-de-Armas *et al.* [23] have simulated in the framework of time-dependent density functional theory the adsorption of different dye molecules on titanium oxide clusters of several sizes (U${}_{n}$[2H${}_{2}$O], where U${}_{n}$ is a TiO${}_{2}$ unit with n∈{2,3,6,9,15,38}). From these calculations, they suggest that, although a TiO${}_{4}$H${}_{4}$ cluster forms a minimal model that can account for the qualitative features of the absorption spectra, it might be limited in describing the electronic structure of the system. However, the electronic structure of the adsorbed dye molecules is seen to change with the cluster sizes. Their results propose a unit (TiO${}_{2}$)${}_{6}$ as the smallest nano-crystallite model able to reproduce semi-quantitatively all features in the electronic structure of the systems. They conclude that the larger clusters do not change much the estimated red shift, which is found in excellent agreement with the experiment.

From a dynamical perspective, the injection process has also been studied both theoretically and experimentally. Wachtweitl and co-workers [15] found that electronic injection proceeds on the femtosecond timescale, with an injection time ranging from 6 fs for colloidal nano-crystallites to 60 fs for titanium oxide thin films. Similar injection timescales were later obtained by Batista and co-workers [24], who investigated the electron transfer process in catechol/TiO${}_{2}$-anatase by means of DFT molecular dynamics simulations. Theoretical simulations at the time-dependent density functional tight-binding level have demonstrated that the injection process in alizarin-TiO${}_{2}$ is of indirect nature [7], meaning that oscillations of the electron density between the dye and the nano-crystallite are observed before irreversible injection happens within tens to hundreds of femtoseconds [25,26]. In this case, the rates were obtained both by simple first-order kinetic modeling and by time-propagation of the atomic orbital populations initiated by δ-pulse excitations. Li *et al.* [26] have investigated the effect of nuclear dynamics on the injection process by means of the multi-layer multi-configuration time-dependent Hartree method. The coupled nuclear-electron dynamics was treated at the harmonic level within the one-electron, molecular orbital picture, and the vibration-electron coupling constants were related to the reorganization energy upon electron transfer. Using Löwdin analysis to generate localized initial conditions on the dye, it was observed that electronic injection proceeds indirectly on a timescale in good agreement with the experiment, while vibrational effects were seen to cause only small oscillations in the donor population. Duncan and Prezhdo [20] have also investigated the injection process using nonadiabatic molecular dynamics simulations, where they found that vibration-electron coupling plays a significant role in the photoinduced electron transfer between alizarin and a hydrated Ti${}^{4+}$ ion. Using a similar methodology at the periodic density functional theory level, the experimental timescale of 6 fs for the injection process was reproduced by the same authors [21]. Interestingly, this timescale was experimentally found to be much longer for thin films, about 60 fs, which should be the situation described by the slab model. This discrepancy between theory and experiment was also pointed out by Li *et al.* [26]. For all that is known about the injection process, time-resolved information about the flow of electron density during the photoinduced charge migration remains unavailable.

In this work, we investigate the injection process from alizarin to TiO${}_{2}$ in colloidal form from a purely electronic perspective. This assumption is justified since the vibrational effects have been shown in other works to induce only a marginal effect within the few femtoseconds of the process [8,25]. As in previous theoretical work [8], we present here a single active electron model based on density functional theory. The evolution of the one-electron density is simulated by solving the time-dependent Schrödinger equation subject to absorbing boundary conditions. Various tools for the analysis of the transient electronic density, such as electronic fluxes and the associated flux density, are used here for the first time. In particular, the electronic flux density is expected to shed light on the mechanisms that delay or accelerate the electronic injection process from the alizarin donor to the semiconductor material. This information could be used to propose new potentially more efficient dyes for the DSSC. In order to benchmark our model systems, we first report on density functional theory (DFT) calculations on the alizarin molecule isolated and adsorbed on TiO${}_{2}$ cluster. Here, geometry optimizations of the dye, TiO${}_{2}$-cluster, and dye-TiO${}_{2}$ complexes in their electronic ground state are performed considering solvent effects to provide a proper description of colloidal nano-crystallites. Second, we explore the optical spectrum of the alizarin isolated and anchored on (TiO${}_{2}$)${}_{15}$ using linear response time-dependent density functional theory. Using a simple δ-pulse excitation from an orbital localized on the dye as an initial condition, the electron injection dynamics is then investigated in real time.

## 2. Theoretical Framework

To investigate ultrafast electron dynamics in dye-sensitized solar cells from a purely *ab initio* perspective, a reduced model where the chromophore is adsorbed on a titanium oxide nano-crystallite is used. The latter can be understood as being a free-standing nanoparticle in solution. Following Sánchez-de-Armas *et al.* [23], who studied the effect of the cluster size on the injection process, we choose a small but suitable nano-crystallite to simulate the optical spectrum and the electron dynamics in model dye-sensitized solar cell:${\left({\mathrm{TiO}}_{2}\right)}_{15}$. The starting geometry for the TiO${}_{2}$ cluster in this study was taken from the literature, and correspond to optimized geometry starting from spherically shaped particles in a polar solvent [27]. Upon evaporation of the solvent, the nano-crystallites are deposited on a substrate that mediate the charge separation, creating a highly corrugated surface [28]. Lacking precise knowledge about the surrounding environment for colloidal nano-crystallite complexes we choose to neglect it altogether and take into account the effect of the solvent directly on the complex. To ensure that no dangling bonds appear, the cluster edges are chosen so that all oxygen and titanium atoms in the nano-crystallite have appropriate coordination numbers (2 and 4, respectively).

### 2.1. Static Calculations

**Figure 1:**
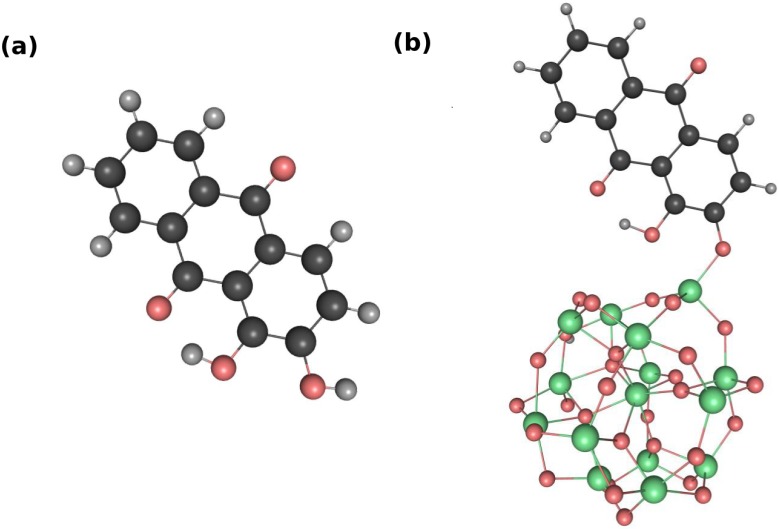
Optimized molecular structure of (**a**) the isolated alizarin dye, (**b**) the alizarin-(TiO${}_{2}$)${}_{15}$ complex. The red, green, black, and grey beads represent oxygen, titanium, carbon, and hydrogen atoms, respectively.

The electronic properties of an alizarin/TiO${}_{2}$ complex are investigated by means of density functional theory (DFT). The method has been shown to yield accurate energies and geometries for similar systems using a variety of exchange-correlation functionals [29,30,31]. In the present work, all density functional calculations have been performed with the program Gaussian 09 [32]. The structure optimizations of the alizarin and (TiO${}_{2}$)${}_{15}$, as well as alizarin-(TiO${}_{2}$)${}_{15}$ neutral system (Figure 1a,b), are performed by using the Perdew-Burke-Ernzerhof (PBE) exchange-correlation functional [33], with the 6-31G** basis set, within the generalized gradient approximation (GGA). This method is known to yield accurate structures while underestimating the HOMO-LUMO energy gap. To check the adequacy of the GGA functional to reproduce the electronic properties of the alizarin and alizarin-(TiO${}_{2}$)${}_{15}$, we perform additional calculations using Becke three-parameter-Lee-Parr-Yang (B3LYP) functional [34], with the LANL2DZ basis set [35] on Ti atoms, and a 6-31G** basis set on all other atoms. It has been shown that the B3LYP/6-31G** level of theory is suitable to reproduce the experimental photochemical and photophysical properties of alizarin, as reported recently [17]. Solvation effects were simulated by the Polarizable Continuum Model (PCM) [36], using acetonitrile as solvent which is commonly used in DSSC [37]. The molecular structures were fully optimized without symmetry constraints and frequency analyses were performed to confirm that all optimized structures are stationary minima points using both functionals. The hybrid functional including exact exchange in the functional often yields better thermochemical properties. Here, we explicitly avoid using long-range corrected functionals, which correct for the overestimation of the long-range Coulomb interaction in common functionals. By doing so, we are able to mimic the electrostatic effect of a larger TiO${}_{2}$ nano-crystallite on the adsorbed alizarin using only small clusters. To simulate the absorption spectra of the dye-sensitized nano-crystallite, the excitation energies and oscillator strengths of the alizarin and alizarin-(TiO${}_{2}$)${}_{15}$ were estimated by using linear response time-dependent density functional theory (TDDFT) [38], employing the same functional and basis sets as for the geometry optimizations. This method is similar to the configuration interaction singles (CIS) method [20], where coupled electron-hole pairs from a reference ground state configuration represent approximations to the true electronic excited states. TDDFT has the main advantage that the electrons and holes, obtained from the occupied and virtual orbitals, are received from a DFT reference that includes part of the correlation energy, whereas CIS is based on a Hartree-Fock reference. The quality of the reference configuration can be evaluated in first approximation by comparing the size of the HOMO-LUMO gap, which is found to be too small at the PBE level and too large at the Hartree-Fock level in comparison with experimental excitation energies, but quite reasonable at the B3LYP level. This qualitative improvement is not solely due to the difference in the computed orbitals but in the form of the effective Hamiltonian used in DFT. In previous work, it was shown that TDDFT/B3LYP yields accurate spectra for free and bound alizarin and catechol, and we choose this same combination of methods in the present work.

To summarize, the cluster is chosen so that there are no dangling bonds and no saturation is needed. Further, the structure represents the highly corrugated surface obtained from colloidal deposition techniques. In this case, the exact structure of the environment is unknown. Point charge embedding of the cluster is expected to change the electronic structure inside the cluster and lead to different band alignment between alizarin and the cluster. Long-range Coulomb interaction can stabilize charge transfer states, and it is expected that the electronic structure of such an embedded cluster would be better described using long-range corrected density functionals (e.g., CAM-B3LYP or LC-PBE). The functional chosen in the present work, B3LYP, overstabilizes charge transfer states, thus mimicking the effect a large number of point charges surrounding the cluster. By doing so, the conduction band of the TiO${}_{2}$ is properly aligned with the LUMO level of the acceptor. This was confirmed by inspection of the orbital hybridization and by comparing the optical spectrum with experiment.

### 2.2. Dynamical Calculations

Despite yielding important information about the spectral characteristics and the band alignment in the donor/acceptor pair, static and response calculations cannot provide insight in the time-evolution of transient electronic densities. In the following, we present a single active electron model and a toolset for its analysis, which reveal certain aspects of the intricate dynamics of electrons in dye-sensitized solar cells upon light excitation. Our framework differs substantially from that one implemented by Meng and Kaxiras, the real-time TDDFT for excited states dynamics simulations [39], in that our formalism neglects the effects of nuclear motion. Since no bond is broken and/or formed during the injection process, the nuclei stay close to their equilibrium positions and their motion only averages the electron density over several nuclear configurations, *i.e.*, the electron dynamics is effectively driven by the coherences between the virtual orbitals and not by the nuclear motion. The effect of the nuclear motion on the electronic probability and flux densities has been previously studied carefully in excited states of H${}_{2}$ [40]. The injection timescale studied here defines an electron transfer and not an electron migration process, which would require treatment of concerted electron-nuclear dynamics.

#### 2.2.1. A Single Active Electron Model

Retaining the one-electron, DFT-based picture used in the previous section for the static characterizations, we choose a simple representation for the time-dependent one-electron wave function as a linear combination of virtual orbitals

(1) $$\text{Ψ}\left(r,t\right)=\sum_{r\in \mathrm{virtual}}D_{r}(t){\text{φ}}_{r}\left(r\right)$$

with expansion coefficients $D_{r}\left(t\right)$. Here, we consider only the contribution of a complete set of virtual Kohn-Sham orbitals ${\text{φ}}_{r}\left(r\right)$ obtained from a ground state DFT calculation, as described in the previous section. This choice is equivalent to an expansion in the basis of singly excited determinants obtained from a B3LYP ground state calculation. The electronic wave function is simply related to the following time-dependent one-electron density

(2) $$\begin{array}{cc} \text{ρ}(r,t) & =\text{Ψ}\left(r,t\right){\text{Ψ}}^{†}\left(r,t\right)\\ & =\sum_{r,s\in \mathrm{virtual}}D_{r}\left(t\right){D_{s}}^{†}\left(t\right){\text{φ}}_{r}\left(r\right){\text{φ}}_{s}\left(r\right)\; \end{array}$$

The time-dependence of the expansion coefficients satisfies the time-dependent Schrödinger equation, which defines the evolution of the electronic density uniquely. Assuming the electronic Hamiltonian to be diagonal in the chosen one-electron basis, ${\hat{H}}_{\mathrm{el}}{\text{φ}}_{r}\left(r\right)={\text{ε}}_{r}{\text{φ}}_{r}\left(r\right)$, the equations of motion for the coefficients of the electronic wave packet in Equation (1) can be integrated analytically

(3) $$D_{r}\left(t\right)=D_{r}\left(t=0\right)\exp\left(-ı{\text{ε}}_{r}t/\hbar \right)\;$$

Thus, apart from the Kohn-Sham orbital energies obtained from a single static calculation, only the initial conditions are required to define the coherent electron dynamics at all times.

For the light-induced injection process investigated here, we consider that the electronic density is excited from an orbital mostly localized on the donor to the space of virtual orbitals. This is a reasonable choice since the light absorption will effectively happen at the interface, with the incident radiation efficiently damped inside and below the nano-crystallite. Further, the large band gap in TiO${}_{2}$ will also prevent excitations localized in the cluster alone. Upon inspection of the orbitals at the B3LYP level of theory, it appears that the HOMO orbital (H) is the highest energy orbital exhibiting strong localization on the chromophore. Thus, it provides a suitable initial configuration for the excitation, *i.e.*, only configurations involving transitions H→r need to be considered. As will be argued below, similar conclusions can be drawn from other similar choices of initial configuration, *i.e.*, promoting electrons from many occupied states simultaneously. For dye-sensitized solar cell applications, the range of relevant orbitals included in the basis should cover the full visible solar spectral bandwidth – up to 6 eV above the ground state energy. In the following, we have thus adopted an initial state considering only configurations involving the promotion of an electron to this energy-selected range of virtual orbitals.

For low-energy light excitations in nanostructures of moderate dimensions, photon absorption can be understood as the semi-classical coupling between an external electric field and the internal dipole moment. Keeping in mind that the excitation source (either sunlight or strongly broadened femtosecond laser pulse) has a large spectral bandwidth, it is appropriate to consider a δ-pulse excitation for which all available frequencies are excited simultaneously. Since we have no prior knowledge of the orientation of the nano-crystallite, it is further important to take into account all three component of the field-dipole interaction. The initial coefficient of orbital *r* is thus chosen proportional to the Golden rule expression for the transition probability

(4) $$D_{r}\left(t=0\right)\propto \sum_{a}\left({є}_{r}-{є}_{a}\right)\sum_{q=x,y,z}{\left|{\text{μ}}_{ar}^{\left(q\right)}\right|}^{2}\;$$

where the transition dipole elements are given by

(5) $${\text{μ}}_{ar}^{\left(q\right)}=-e\langle {\text{φ}}_{a}|q|{\text{φ}}_{r}\rangle \;$$

Here, the index *a* denotes an occupied orbital. The coefficients are uniquely defined by imposing the normalization condition to the wave packet at all times, 〈Ψ(t)|Ψ(t)〉=1. This choice of initial condition is equivalent to promoting the initial occupied ${\text{φ}}_{a}$ orbitals onto the space of active virtual orbitals.

The nano-crystallites used in the present work are rather small, and an initially localized wave packet will spread rapidly to reach the edges and be reflected back in the cluster. According to Equation (3), the coherent dynamics will lead to fast recurrences and quasi-recurrences in the electron dynamics. The true physical system will not see those recurrences, as the coupling to the environment will favor electron diffusion over coalescence of the electronic wave packet on ultrafast timescales. This observation is valid for both nano-crystallites in solution, which are sometimes stabilized by an organic coating layer, and cluster embedded in a larger TiO${}_{2}$ environment. To avoid artificial reflection at the end of the cluster, we thus impose absorbing boundary conditions using a complex absorbing potential and modify the system Hamiltonian as ${\hat{H}}_{\mathrm{el}}\to {\hat{H}}_{\mathrm{el}}-ı{\hat{W}}_{\mathrm{CAP}}$. It was demonstrated that resolving the contribution of the absorbing potential in an atom-centered basis requires a very large number of auxiliary functions distributed along molecular bonds [41]. The computation cost of this procedure is prohibitively high even for small molecules. To circumvent this problem, we define the absorbing potential using the projector formalism

(6) $${\hat{W}}_{\mathrm{CAP}}={\text{γ}}_{\mathrm{CAP}}{\hat{P}}_{\mathrm{sink}}\;$$

where ${\text{γ}}_{\mathrm{CAP}}$ is a user-defined parameter which should remain small. It follows that the absorbing potential only contributes as diagonal corrections to the electron dynamics

(7) $$D_{r}\left(t\right)=D_{r}\left(t=0\right)\exp\left(-ı{\text{δ}}_{r}t/\hbar -{\text{Γ}}_{r}t/2\right)\;$$

where the relaxation rate ${\text{Γ}}_{r}=\langle {\text{φ}}_{r}|{\hat{W}}_{\mathrm{CAP}}|{\text{φ}}_{r}\rangle$ leads to the non-unitary evolution of the system.

The projector on the sink, ${\hat{P}}_{\mathrm{sink}}$, is defined as the sum of projectors on the oxygen atoms at the edges of the cluster, as they are providing the link between the nano-crystallite and the surroundings. This approach is similar to the one followed by Li *et al.* [25,26]. In the MO-LCAO (Molecular Orbital— Linear Combination of Atomic Orbitals) ansatz, the Kohn-Sham orbitals ${\text{φ}}_{r}\left(r\right)$ are expanded in a set of atomic orbitals ${\text{χ}}_{\nu }^{A}\left(r-R_{A}\right)$

(8) $${\text{φ}}_{r}\left(r\right)=\sum_{A}\sum_{{\text{ν}}_{A}}C_{r{\text{ν}}_{A}}{\text{χ}}_{r{\text{ν}}_{A}}\left(r-R_{A}\right)\;$$

where $C_{r{\text{ν}}_{A}}$ are the MO coefficients and the sums run over all atomic orbitals centered on atom *A*. Exploiting this structure, the relaxation rates take the following simple form

(9) $$\begin{array}{cc} {\text{Γ}}_{r} & ={\text{γ}}_{\mathrm{CAP}}\langle {\text{φ}}_{r}|{\hat{P}}_{\mathrm{sink}}|{\text{φ}}_{r}\rangle \\ & ={\text{γ}}_{\mathrm{CAP}}\sum_{\kappa \in O}\sum_{{\text{ν}}_{\kappa }}\sum_{A}\sum_{{\text{ν}}_{A}}C_{r{\text{ν}}_{\kappa }}C_{r{\text{ν}}_{A}}S_{{\text{ν}}_{\kappa }{\text{ν}}_{A}} \end{array}$$

with $S_{{\text{ν}}_{\kappa }{\text{ν}}_{A}}=\langle {\text{χ}}_{{\text{ν}}_{\kappa }}|{\text{χ}}_{{\text{ν}}_{A}}\rangle$ a matrix element of the atomic orbital overlap matrix.

#### 2.2.2. An Electronic Flux Analysis Toolset

Various tools can be used to understand the intricate electron dynamics in the dye-sensitized nano-crystallites described above. We advocate using the electronic flux density and derived quantities to shed light on the ultrafast charge injection process from the dye to the acceptor. Recasting the time-dependent electronic Schrödinger equation, Equation (3), for the electronic density leads to the celebrated continuity equation [42]

(10) $$\frac{\partial }{\partial t}\text{ρ}(r,t)=-\nabla \cdot j\left(r,t\right)\;$$

where $j_{el}\left(r,t\right)$ is the electronic flux density. The flux density is a vector field that yields information about the evolution of the electronic density elements, which is interpreted as a probability density fluid. Making the simple choice of zero gauge, the flux density takes the form

(11) $$j\left(r,t\right)=-\frac{\hbar }{m_{e}}Im\left[\text{Ψ}\left(r,t\right)\nabla {\text{Ψ}}^{†}\left(r,t\right)\right]\;$$

Using the expansion in the basis of Kohn-Sham orbitals, Equation (1), the evolution of the electronic flux density is seen to be solely driven by the time-dependence of the coefficients

(12) $$j\left(r,t\right)=-\frac{\hbar }{m_{e}}\sum_{r\neq s}Im\left[D_{r}\left(t\right){D_{s}}^{†}\left(t\right)\right]\left({\text{φ}}_{r}\left(r\right)\vec{\nabla }{\text{φ}}_{s}\left(r\right)\right)\;$$

The position of the nodes can be inferred from the interference patterns appearing in the equation due to the propagation of the coefficients $D_{r}$. Hence, the flux density yields information about the evolution of both the electronic density and the associated wave function. Note that, since the density is subject to a non-unitary dynamics, the electronic flux will vanish at longer times. The electronic flux density has been used to study the electronic motion in different scenarios, e.g., the $\text{π}-{\text{π}}^{*}$ transition in ethylene and n−π transitions in formaldehyde [43], electron flux in formic acid dimer on the occasion of double proton transfer [44], polarization of the H${}_{2}$ bond by short laser pulses [40], and in the coherent electron-nuclear motion of a vibrating H${}_{2}^{+}$ molecular ion [45,46]. Here we calculate the electronic flux density for an injection process for the first time.

To analyse the time- and space-resolved evolution of the electronic density, we resort to the projector formalism. For a given molecular structure, the space is partitioned between atoms or fragments using the Voronoi formalism, which assigns each point in space to the closest-lying atom or fragment. This type of geometric partitioning is used for the definition of a so-called Voronoi Deformation Density (VDD) charge, which has been shown to yield reliable predictions of atomic charges in a large number of molecules with faster convergence with respect to the basis set size as compared to, e.g., the Mulliken analysis [47]. Accordingly, we define the number of electrons $n_{V}\left(t\right)$ in a chosen volume at any given time by

(13) $$n_{V}\left(t\right)=\int dr\;{\hat{P}}_{V}\text{ρ}\left(r,t\right)\;$$

where ${\hat{P}}_{V}$ is a projector on the Voronoi cell containing the selected atoms of the fragment. The projector formalism can be used to strictly define complementary tools for the analysis of the dynamics. The electronic flux ($F_{V}\left(t\right)$), or the flow of electrons in and out of a chosen integration volume, can be written as

(14) $$F_{V}\left(t\right)=\int dr\;\frac{\partial {\text{ρ}}_{V}\left(r,t\right)}{\partial t}\;$$

Here, ${\text{ρ}}_{V}\left(r,t\right)={\hat{P}}_{V}\text{ρ}\left(r,t\right)$ is the projection of the one-electronic density on the Voronoi cell volume. Integrating the electronic flux in the Voronoi cell over time gives access to the so-called electron yield

(15) $$\begin{array}{cc} Y_{V}\left(t\right) & =\int_{0}^{t}dt^{\prime }\;\int dr\;\frac{\partial {\text{ρ}}_{V}\left(r,t^{\prime }\right)}{\partial t^{\prime }}\\ & =n_{V}\left(t\right)-n_{V}\left(0\right)\; \end{array}$$

which can be simply related to the number of electrons in the chosen volume. These tools yield information about the instantaneous and the total contributions of a fragment to the electron injection process, respectively.

In the present work, post-processing of the static DFT calculations was performed using orbkit [48], which is an in-house python program for the grid-based representation of one-electron quantities. In the present case, these include the one-electron densities, the orbitals, the electronic fluxes, and the flux densities. The transition dipole moments and the integrals required to define the initial conditions, as well as the point-wise definition of the Voronoi cells and the underlying integrals were also performed with orbkit [48]. The dynamics was represented using the ZIBAmira visualization program [49].

## 3. Results and Discussion

### 3.1. Static Considerations

The optimized structures of the alizarin molecule in the gas phase and adsorbed on the nano-crystallite are shown in Figure 1. It corresponds to a monodentate attachment to the TiO${}_{2}$ cluster, while a bidentate species has been also reported [19]. Interestingly, the type of coordination between the alizarin and the TiO${}_{2}$ cluster seems to play secondary role in injection process (compare Refs. [19] and [23]). It can be seen that, upon optimization, the initially spherical structure of the titanium oxide nano-crystallite is well preserved for the small (TiO${}_{2}$)${}_{15}$ cluster. For larger clusters, the spherical structure has been observed to become significantly distorted [23]. We attribute this difference to the presence of the polar solvent, which seems to favor structures where oxygen atoms point outside the cluster. The large clusters possess enough flexibility to facilitate the rotation of the oxygen atoms outwards, while the relatively rigid small cluster used here approximately retains the spherical symmetry of the initial structure. This observation is in line with the findings of Sánchez *et al.* [23]

**Figure 2:**
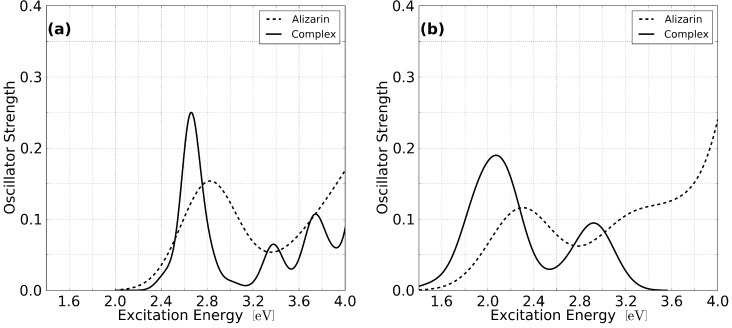
Optical spectra for isolated alizarin and the alizarin-(TiO${}_{2}$)${}_{15}$ complex obtained employing linear response TDDFT and the (**a**) B3LYP and (**b**) PBE functionals. The spectra have been broadened using Lorentzians with width of δ=0.50 (isolated alizarin) and δ=0.20 (alizarin-(TiO${}_{2}$)${}_{15}$).

Table 1 shows the comparison between TDDFT simulations with different functionals and experimental results for the vertical electronic transitions in free alizarin and alizarin anchored to the nano-crystallite. The lowest energy band for the free alizarin is observed at 2.82 eV and 2.29 eV using B3LYP and PBE, respectively. As in other theoretical work where the solvent effects were neglected, the B3LYP functional gives a better agreement with experiment, with the first absorption peak found at 2.88 eV. Both functionals have an important component coming from the transition from the HOMO to the LUMO which shows similar oscillator strength (not reported, see spectrum in Figure 2).

**Table 1:** Calculated (Calc.) and experimental (Exp. [14]) excitation energies in eV, active molecular orbitals (MO) and their contributions in %. Top panel: time-dependent density functional theory (TDDFT)/Becke three-parameter-Lee-Parr-Yang (B3LYP); bottom panel: TDDFT/Perdew-Burke-Ernzerhof (PBE).

Alizarin			Alizarin-(TiO${}_{2}$)${}_{15}$		
Calc.	Exp. [14]	MO (%)	Calc.	Exp.	MO (%)
2.82	2.88	HOMO→LUMO (97.7)	2.66 3.35	2.47 3.54	HOMO→LUMO+2 (95.5)
					HOMO-1→LUMO+1 (35.3)
					HOMO-1→LUMO+2 (25.8)
					HOMO-1→LUMO+5 (6.9)
					HOMO→LUMO+4 (11.7)
			3.74	3.54	HOMO-4→LUMO+2 (43.9)
					HOMO-4→LUMO+1 (9.4)
					eHOMO-4→LUMO+5 (9.5)
					HOMO→LUMO+20 (7.8)
					HOMO→LUMO+22 (9.7)
2.29 3.28	2.88 3.82	HOMO→LUMO (95.1) HOMO-4→LUMO (58.3) HOMO→LUMO+1 (35.0)	2.16	2.47	HOMO→LUMO+2 (25.6)
					HOMO→LUMO+4 (11.3)
					HOMO→LUMO+11 (17.6)
					HOMO→LUMO+12 (24.0)
			2.91	3.54	HOMO-5→LUMO+3 (18.9)
					HOMO-6→LUMO+2 (7.2)
					HOMO-4→LUMO+2 (10.7)
					HOMO-6→LUMO+4 (6.5)
					HOMO-4→LUMO+7 (11.7)
					HOMO→LUMO+26 (17.7)

**Figure 3:**
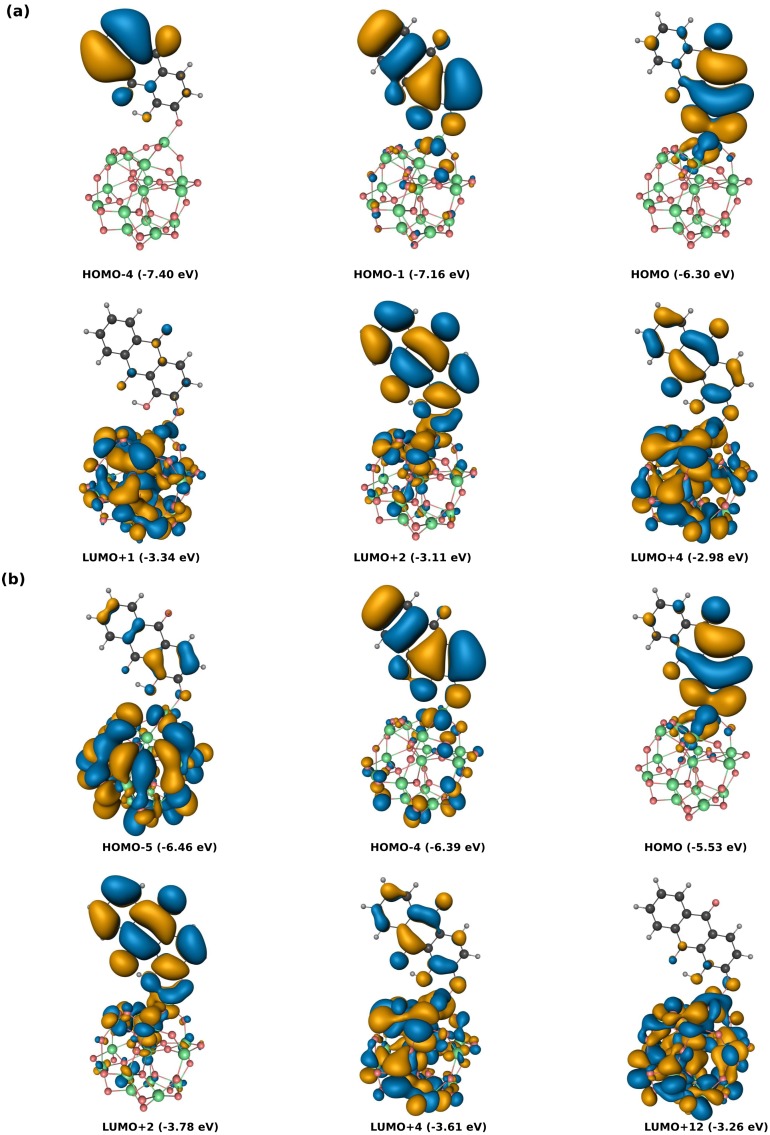
Dominant molecular orbital contributions to the excitations of selected low energy bands in the alizarin-(TiO${}_{2}$)${}_{15}$ complex obtained using (**a**) B3LYP and (**b**) PBE. Isosurface value is set to $0.0675\;{\text{Å}}^{-3}$.

The isosurfaces of selected MOs involved in the simulated transitions of the alizarin-(TiO${}_{2}$)${}_{15}$ system are presented in Figure 3. With both functionals, the occupied orbitals are observed to be localized mainly over the alizarin framework, as is the case for the LUMO+2, but the latter orbital has a larger spread over the semiconductor cluster. The HOMO-1 obtained at the B3LYP level correlates well with the lower-lying HOMO-4 from the PBE simulation. The LUMO+4 is also strongly represented in many transitions at low energy, and is seen to be similar for both functionals. Other notable contributions come from either delocalized orbitals (e.g., HOMO-5 for PBE) or orbitals localized on the nano-crystallite (e.g., LUMO+1 for B3LYP or LUMO+12 for PBE), which have widely different energetic properties with both functionals.

Interestingly, the first band in the alizarin-(TiO${}_{2}$)${}_{15}$ spectrum originates from a single transition, HOMO→LUMO+2, when employing B3LYP (see Table 1). This is in stark contrast with the results obtained at the PBE level, where the band is composed of several transitions from the HOMO to different virtual orbitals localized in the nano-crystallite. Nonetheless, the splitting of the localized excitation to multiple virtual orbitals on the cluster is also observed for higher excited states, both at the PBE and B3LYP level. The character of many dye-localized orbitals involved in the optical transitions is very similar for both functionals, while their contribution to the excited states varies widely. From Figure 3b and the data reported in the Table 1, it can be inferred that both functionals yield a sensible model for local transport of electron in a dye-sensitized TiO${}_{2}$ solar cell. The first transition at the B3LYP level involves occupied orbitals localized on the dye to orbitals that are well hybridized with those of the nano-crystallite, a fact that indicates strong coupling between the dye and TiO${}_{2}$ and proper energetic alignment of the donor and acceptor states. Whereas similar trends are observed at the PBE level, more spurious transitions between orbitals localized in the cluster appear in the bands. The energies obtained for the optical spectrum should also be taken with care, especially at the PBE level, since linear response TDDFT depends strongly on the overlap between initial and final orbitals to yield meaningful results. This problem is mitigated in B3LYP by inclusion of exact exchange, which is non-local and renders less sensitive to the overlap between the reference and the virtual orbitals. The orbitals involved in the lowest optical bands of the complex being similar for both functional, the comparison of transition energies with experiment yields a better criterion to evaluate the suitability of the underlying functional. Hence, it follows that TDDFT/B3LYP is a suitable level of theory for the current investigations, while care should be taken in over-analyzing results obtained using the PBE functional. This conclusion is in line with results obtained by others for chromophores on titanium oxide [3].

The simulated spectra from TDDFT calculations for the free alizarin and the adsorbed alizarin to the cluster are shown in Figure 2. The spectra are smoothed using Lorentzian broadening with a width of σ=0.50 (isolated alizarin) and σ=0.20 (alizarin-(TiO${}_{2}$)${}_{15}$). In this figure, the expected red-shift of the alizarin lowest energy band induced by its coupling to the TiO${}_{2}$ cluster is observed.

The spectrum of the free molecule is composed of a single peak at the B3LYP level in the range depicted, while a second peak at 3.28 eV is observed at the PBE level and is attributed to the combination of local excitations HOMO-4→LUMO and HOMO→LUMO+1. This second band is experimentally observed and suspiciously absent from the TDDFT/B3LYP spectrum. Inspection of the frontier orbitals reveals that the HOMO of the gas phase molecule retains its character in the complex, while the LUMO is shifted higher in the density of states at the LUMO+2 level. This ensures a better alignment of the latter with the orbitals of the (TiO${}_{2}$)${}_{15}$ nano-crystallite. Whereas the PBE spectrum for the adsorbed species is also seen to be red-shifted, the position of the bands and the number thereof are in poor agreement with experiment in the depicted window.

### 3.2. Dynamical Considerations

In order to simulate the injection process in real time, *i.e.*, the electron transfer from the alizarin towards the TiO${}_{2}$ cluster, we consider an initial (t=0) electronic wave packet formed by a set of virtual molecular orbitals (MOs) obtained at the B3LYP(6-31G**) level for the alizarin-(TiO${}_{2}$)${}_{15}$ system. The probability amplitude of each virtual MO is determined by the dipole-induced transition with a preselected set of occupied MOs according to the single active electron model (see Equations (1)–(5)). In the following, all simulations presented are performed with a single initial orbital, the HOMO, as the results were only marginally affected by the number of localized occupied MOs in the definition of the initial condition. In order to prevent unphysical reflections of the wave packet, absorbing potentials were applied to the basis functions centered at the oxygen atoms located at the edges of the TiO${}_{2}$ cluster. The relaxation rates of the absorbing potentials were chosen such as the entire dynamics happens in a few tens of femtoseconds, according to experimental evidence [15].

**Figure 4:**
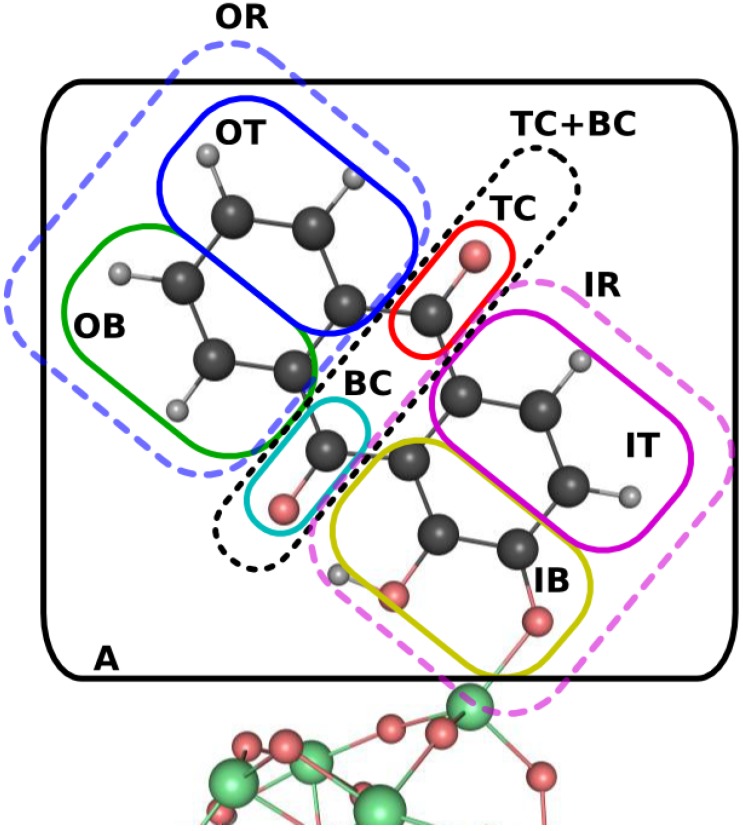
Definition of projectors for the analysis of the electron flux and yield. The boxes define the atoms belonging to a particular fragment, for which a projector is defined using a Voronoi partitioning. The labels OT (IT), OB (IB), and OR (IR) stand for Outer (Inner) Top ring, Bottom ring, and complete Ring, respectively. The carbonyl fragments are labeled TC (**top**) and BC (**bottom**), and the label A refers to the total projector on alizarin.

To understand the flow of electrons using integrated quantities such as the yield and the flux, it is necessary to define the fragments associated with the projector ${\hat{P}}_{V}$ (see Equations (13) and (14)). As depicted in Figure 4, the alizarin unit is decomposed in various overlapping fragments. The smallest fragments are defined as follows: the Outer Top (OT) ring, the Outer Bottom (OB) ring, the Inner Top (IT) ring, the Inner Bottom (IB) ring, the Top Carbonyl (TC), and the Bottom Carbonyl (BC). Larger units divide the dye in the two carbonyls (TC+BC) and two rings: an Outer (OR) and an Inner ring (IR). These volumes are not unique but they are chosen here to reveal important aspects of the electron dynamics: contribution to the electron injection process, synchronicity and symmetry of the electron flow, outcome of the aromaticity, *etc*. The total flow of electrons outside the chromophore can be obtained from the sum of non-overlapping projectors on the molecule, since the Voronoi partitioning scheme is additive by definition.

**Figure 5:**
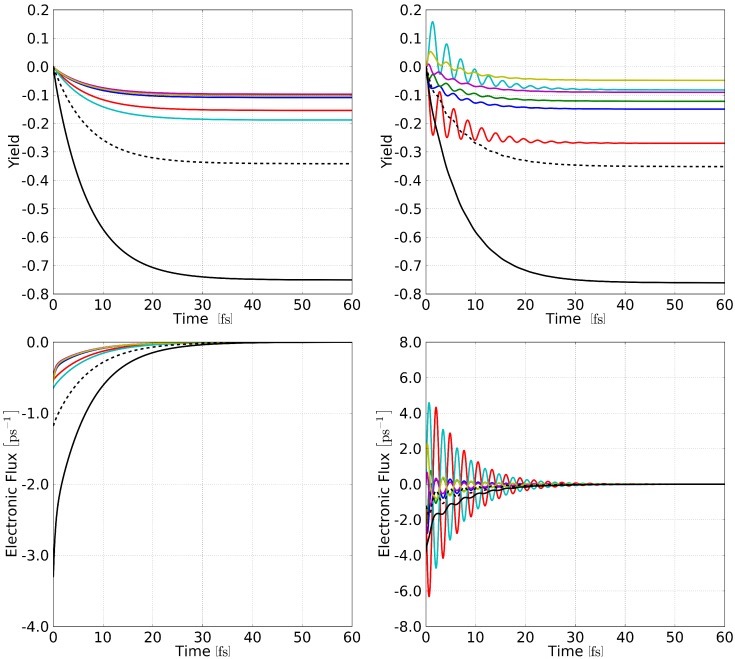
Evolution of the electronic yield, Equation (15), and associated flux, Equation (14), during the charge transfer dynamics from the alizarin towards the TiO${}_{2}$ cluster. Projectors on the Outer Top ring (blue), Outer Bottom ring (green), Inner Top ring (magenta), Inner Bottom ring (yellow), Top Carbonyl (red), and Bottom Carbonyl (cyan) are depicted, as well as the projector on the central unit (dashed) and the complete alizarin (solid black). Left panels: incoherent propagation of the electronic density. Right panels: coherent propagation of the electronic wave packet.

Figure 5 displays the yield, Equation (15), and the electronic fluxes, Equation (14), as a function of time for two different simulations: incoherent (left panels) and coherent (right panels) propagation of the electronic wave packet. The latter simulation represents the δ-pulse initial condition which most resembles femtosecond laser experimental conditions. The former can be assimilated to broadband sunlight excitation, which happens on a much longer timescale than decoherence in the system. The incoherent one-electron density is in this case constructed by neglecting interference effects in Equation (2), $\text{ρ}(r,t)=\sum_{r\in \mathrm{virtual}}{\left|D_{r}\left(t\right)\right|}^{2}{\text{φ}}_{r}^{2}\left(r\right)$, where the coefficients are otherwise propagated analytically using Equation (7). Notice that the charge transfer, here represented by the total number of electrons on the alizarin ($n_{A}\left(t\right)$), mostly happens during the first 10 to 20 fs of the dynamics, where 60% to 70% of the population on the alizarin is lost. Surprisingly the charge transfer from alizarin towards the TiO${}_{2}$ cluster seems to be almost independent on the coherence of the wave packet, with the coherent scenario being marginally faster. The timescale for the injection can be accurately fitted to an exponentially decaying function, yielding a lifetime of 6.77 fs and 6.90 fs in the coherent and incoherent case, respectively. In the asymptotic limit, the dye is seen to contribute to 75.9% (75.0%) of the electron injection process in the coherent (incoherent) scenario. From these small variations, we infer that the charge injection could be accelerated by creating a coherent superposition of many states within a complex with a better band alignment, *i.e.*, with a larger number dye-localized excited states lying energetically in the conduction band of the nano-crystallite. It is worth mentioning that, whereas the timescales extracted from the exponential fit match the experiment remarkably well, the boundary conditions used in the definition of the absorbing potential are orders of magnitude faster.

From the various projectors defined in Figure 4, it is possible to extract information about the electron flow between different sections of the alizarin. Two observations can be immediately made: (1) coherence plays an important role in the final populations of the components of the alizarin, *i.e.*, from which fragment the electron is transferred to the cluster, even if the total population only marginally depends on it; and (2) the short-time population evolution of the two carbonyl groups is strongly affected by the coherence when considered separately, but not when considered as a whole (TC+BC). The loss of population in the different ring fragments (IT, OT, IB, OB) does not surpass the ∼10%, being almost equal for all the fragments in the incoherent propagation. As one would expect from Equation (14), the incoherent flux mirrors the yield dynamics and vanishes at longer time. By construction, the incoherent dynamics exhibits a simple exponential decay behavior of the density localized on each fragment after the initial excitation, as can be seen from both the yields and the electronic fluxes. More importantly, the decay timescale is seen to be the same for all fragments.

The coherent case reveals a slight asymmetry among the ring fragment contributions, where the loss of the population is larger in the OT ring, followed by the OB ring, the IT ring, and, finally, the IB ring. Inspection of the oscillatory patterns reveals that the motion within the outer in inner rings, *i.e.*, from OT to OB and from IT to IB, are synchronous. On the other hand, the dynamics presents a slightly asymmetric electronic flux in the inner ring during the first oscillation period, which could be caused by the instantaneous nature of the chosen excited initial conditions. The electronic flux is the time derivative of the yield and, as such, is more sensitive to small fluctuations of the density. More interesting is the case of the two carbonyl groups in the coherent propagation. During the first 10 fs, the bottom carbonyl group experiences four oscillations in its charge population, gaining around 18% of population during the first ∼2 fs (positive value of the charge transfer), and then experiencing a loss of population until ∼3 fs to return to its original value (zero charge transfer). This process is then repeated until ∼5 fs but with a lower gain at ∼9%. Two further oscillations are then observed in the BC population before 10 fs, after which the population starts to oscillate around negative values. The net electron loss amounts to about 5% by the end of the propagation. The oscillatory effect is not observed in the total population of the alizarin and, therefore, should not be observed in the charge transfer toward the TiO${}_{2}$ nano-crystallite.

Interestingly, the top carbonyl group (TC) experiences the same oscillation pattern as the BC fragment but in opposite phase, while the charge transfer remains negative (loss of electronic population) during the entire dynamics. Note that the gain-loss processes in the two carbonyl groups are mutually correlated, as can be seen from the simple exponential behavior of the TC+BC curve, *i.e.*, one has an effective charge transfer between one carbonyl group towards the other through the rings, which ultimately serve as a bridge between the two carbonyl groups. This analysis of the population suggests that a strong alternating electron flux density should exist between the two carbonyl groups, while the entire alizarin experiences a continuous charge transfer toward the cluster. This behavior can be simply explained in terms of a Rabi oscillation between the two dominant contributions to the initial conditions. The oscillation period of about 3 fs correlates perfectly with the inverse of the transition energy between the two excited electronic states, 2πℏ/(1.47eV)∼2.8 fs.

**Figure 6:**
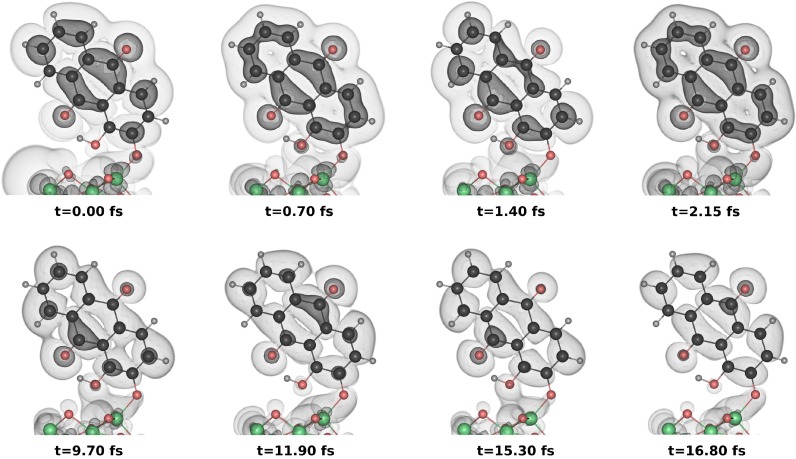
Evolution of the electron density on the alizarin moiety from the coherent wave packet dynamics. The contour values for the dark and light grey contours are chosen as $6.75\cdot 10^{-4}\;{\text{Å}}^{-3}$ and $6.75\cdot 10^{-5}\;{\text{Å}}^{-3}$, respectively.

To gain a qualitative understanding of the dynamics, it is instructive to have a look at the evolution of the one-electron density. Figure 6 shows a few snapshots of the density as the injection reaction proceeds for the coherent simulation. From the initial conditions it can be seen that interferences create an imbalance favoring accumulation of electronic density on the top carbonyl fragment. A slight density asymmetry in favor of the top part is also observed for both the outer and inner rings in the coherent case. This asymmetry is absent from the incoherent density (not shown), and the distribution of the electrons between the two carbonyls or among the rings is more democratic, as can be inferred from the yields, Figure 5. By construction, the incoherent density decays exponentially, and each contribution to the electronic injection corresponds well to the initial density. In the coherent case, the density is seen to wobble along the rings and the carbonyl backbone before vanishing. In the top row of Figure 6, a complete cycle of the Rabi oscillation between the two carbonyl groups is depicted, with turning points at 0.7 fs and 2.15 fs. The asymmetric initial condition is inverted at 1.4 fs and recovered at 2.8 fs, with the former situation presenting a larger asymmetry in the electron distribution between the carbonyl moieties. At longer times (bottom row), when the exponential decay of the electron density becomes visible, the asymmetry at the local maxima (11.9 fs and 16.8 fs) and minima (9.7 fs and 15.3 fs) of the flux is still observed. Although the investigation of the time-dependent density yields some information about the injection process, the detailed contributions to flow of electrons remain elusive using this representation.

**Figure 7:**
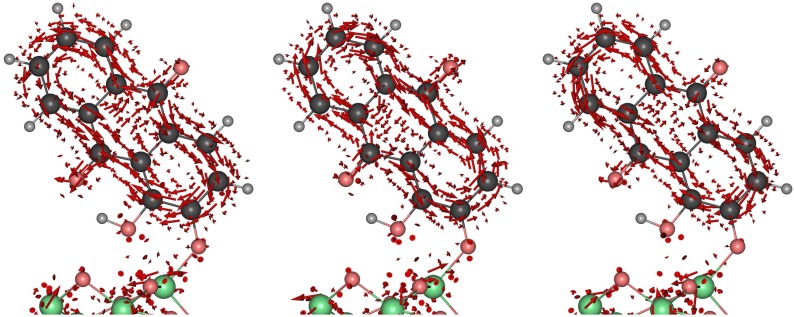
Electronic flux density j(r,t) at t=0.70 fs (**left**), t=2.15 fs (**center**), and t=3.40 fs (**right**). The red arrows describe the instantaneous flow of electronic density. The snapshots were chosen at the turning points in the dynamics, where the electronic density is symmetrically distributed on the molecule (see Figure 6). Electrons are seen to flow from one carbonyl to the other via the aromatic rings. At the t=2.15 fs, the flow direction is reversed.

In order to study how this alternating charge process between the two carbonyl groups happens, *i.e.*, how the electron probability density flows from one of the carbonyl group to the other through the rings, we analyse the electronic flux density j(r,t), which ultimately contains detailed information about the coherence and the electronic probability density ρ(r,t) (see Equations (2), (10) and (11)). A great advantage of the flux density over integrated quantities such as the electronic flux and the associated yield is that it is independent of the definition of the partitioning scheme. Indeed, the choice of projector being not unique, different definition (Löwdin, Mulliken, Voronoi, Bader, Hirschfeld, *etc*.) [47] may yield different pictures for the process. Flux density maps are also independent of the choice of basis set, thus allowing to circumvent that ambiguity, while being limited to situations, where coherences play a role. Figure 7 shows j(r,t) at three different times: at t=0.70 fs when the electrons is transferred from the carbonyl on top to the carbonyl on the bottom, at t=2.15 fs when the transfer process is reversed and at t=3.40 fs completing the first cycle. The flux density maps reveal that the two rings serve as bridges to the electron migration. Note that the partitioning of alizarin in two rings and two carbonyl groups serves only discussion purposes, as the flux density is a vector field yielding information about the flow of electrons at any given point. A circular-like flux density is observed in both rings, which means that at each time, the flux density through the outer carbon atoms of the rings has the same direction as the flux density around the two carbonyl groups (from top to the bottom and vice-versa). On the contrary, the flux between the inner carbon atoms (those ones immediately attached to both carbonyl groups) is pointing in the opposite direction. This result is in agreement with the aromatic character observed in anthraquinone derivatives [50]. Meanwhile, the hydroxyl group close to the cluster surface remains a spectator in the charge transfer process. Finally, the oxygen atom directly attached to the TiO${}_{2}$ cluster serves as a bridge between the alizarin and the cluster. It is observed that, when the current flows from the top to the bottom carbonyl, the electrons flow toward the nano-crystallite, and part of the electron density flows back to alizarin via the same oxygen bridge. Most importantly, the injection process appears to flow *along* the bonds building the bridge between the dye and the cluster, and not through empty space. It is important to note that, while the computation and the representation of the flux density maps may appear cumbersome, they yield valuable information about the electron flow with both spatial and temporal resolution, while alleviating the requirement of arbitrary partitioning schemes.

## 4. Conclusions

In conclusion, we have studied the ultrafast electron transfer dynamics in alizarin adsorbed to a colloidal titanium oxide nano-crystallite using a one-electron, time-dependent density functional theory ansatz. The optical spectra were simulated in linear response, revealing the local character of the excitation for the first few excited states. Our optical spectral for isolated alizarin shows a low energy band at 2.82 eV calculated with B3LYP functional, which is in agreement with the experimental value (2.88 eV), while the value obtained with PBE functional underestimated the transition energy. The superiority of the B3LYP over the PBE functional was underlined by the semi-quantitative agreement of the spectrum with experiment. Qualitative analysis of the orbitals involved in the low-lying bands revealed that, apart from the important contributions from the orbitals localized on the dye, many orbitals localized on the nano-crystallite and other delocalized over the complex play a significant role.

Accordingly, the dynamical simulations were performed using a large basis of virtual orbitals, instantaneously excited via a semi-classical dipole mechanism. The injected electron was absorbed at the end of the numerical grid using a simple local imaginary potential. To reveal the details of the ultrafast injection process, a variety of quantum mechanical analysis tools based on the Voronoi projector formalism and derived from the electronic flux density were introduced. Integrated quantities such as the electronic flux and the electron yield proved useful to extract quantitative information about the flow of electrons and the relative contribution of different fragments to the injection. Incoherent propagation of the density was found to behave much more symmetrically than the coherent propagation of the electronic wave packet, for which Rabi oscillations between the two carbonyl groups were observed.

To avoid resorting to the projector formalism and thereby alleviating their ambiguous definition, we investigated the flux density maps during the reaction. In particular, it was found that the carbonyl groups play an important role in the charge transfer process, while the aromatic rings remain mostly spectator, merely mediating the electron transfer from one carbonyl to the other. Further, although the environment surrounding the two aromatic rings is quite different, the Rabi oscillations within the dye-molecule complex were found to be symmetric and synchronous at all times.

We hope that the formalism presented, namely the analysis of the probability density ρ(r,t) together with its counter part the electronic flux density j(r,t), will serve theoreticians in the study of electron transfer processes in scenarios similar to the one investigated here.
