# Correction: Gomez, T., *et al.* Imaging the Ultrafast Photoelectron Transfer Process in Alizarin-TiO_2_. *Molecules* 2015, *20*, 13830–13853

**DOI:** 10.3390/molecules201019027

**Published:** 2015-10-20

**Authors:** Tatiana Gomez, Gunter Hermann, Ximena Zarate, Jhon Fredy Pérez-Torres, Jean Christophe Tremblay

**Affiliations:** 1 Dirección de Postgrado e Investigación, Universidad Autónoma de Chile, Llano Subercaceaux 2801, San Miguel, Santiago, Chile; E-Mail: ximena.zarate@uautonoma.cl; 2 Institut für Chemie und Biochemie, Freie Universität Berlin, Takustraße 3, 14195 Berlin, Germany; E-Mails: gunter.hermann@fu-berlin.de (G.H.); jperez@zedat.fu-berlin.de (J.F.P.-T.)

The author wishes to make the following correction to this paper [1]. Due to mislabeling, please replace:

**Table 1:** Calculated (Calc.) and experimental (Exp. [14]) excitation energies in eV, active molecular orbitals (MO) and their contributions in %. Top panel: time-dependent density functional theory (TDDFT)/Becke three-parameter-Lee-Parr-Yang (B3LYP); bottom panel: TDDFT/Perdew-Burke-Ernzerhof (PBE).

Alizarin			Alizarin-(TiO_2_)_15_		
Calc.	Exp. [14]	MO (%)	Calc.	Exp.	MO (%)
2.82	2.88	HOMO→LUMO (97.7)	2.66 3.35	2.47 3.54	HOMO→LUMO+2 (95.5)
					HOMO-1→LUMO+1 (35.3)
					HOMO-1→LUMO+2 (25.8)
					HOMO-1→LUMO+5 (6.9)
					HOMO→LUMO+4 (11.7)
			3.74	3.54	HOMO-4→LUMO+2 (43.9)
					HOMO-4→LUMO+1 (9.4)
					eHOMO-4→LUMO+5 (9.5)
					HOMO→LUMO+20 (7.8)
					HOMO→LUMO+22 (9.7)
2.29 3.28	2.88 3.82	HOMO→LUMO (95.1) HOMO-4→LUMO (58.3) HOMO→LUMO+1 (35.0)	2.16	2.47	HOMO→LUMO+2 (25.6)
					HOMO→LUMO+4 (11.3)
					HOMO→LUMO+11 (17.6)
					HOMO→LUMO+12 (24.0)
			2.91	3.54	HOMO-5→LUMO+3 (18.9)
					HOMO-6→LUMO+2 (7.2)
					HOMO-4→LUMO+2 (10.7)
					HOMO-6→LUMO+4 (6.5)
					HOMO-4→LUMO+7 (11.7)
					HOMO→LUMO+26 (17.7)

With this corrected table: 

**Table 1:** Calculated (Calc.) and experimental (Exp. [14]) excitation energies in eV, active molecular orbitals (MO) and their contributions in %. Top panel: time-dependent density functional theory (TDDFT)/Becke three-parameter-Lee-Parr-Yang (B3LYP); bottom panel: TDDFT/Perdew-Burke-Ernzerhof (PBE).

Alizarin			Alizarin-(TiO_2_)_15_		
Calc.	Exp. [14]	MO (%)	Calc.	Exp.	MO (%)
2.82	2.88	HOMO→LUMO (97.7)	2.66	2.47	HOMO→LUMO+2 (95.5)
			3.35	3.54	HOMO-1→LUMO+1 (35.3)
					HOMO-1→LUMO+2 (25.8)
					HOMO-1→LUMO+5 (6.9)
					HOMO→LUMO+4 (11.7)
			3.74	3.54	HOMO-4→LUMO+2 (43.9)
					HOMO-4→LUMO+1 (9.4)
					HOMO-4→LUMO+5 (9.5)
					HOMO→LUMO+20 (7.8)
					HOMO→LUMO+22 (9.7)
2.29	2.88	HOMO→LUMO (95.1)	2.16	2.47	HOMO→LUMO+2 (25.6)
3.28	3.82	HOMO-4→LUMO (58.3)			HOMO→LUMO+4 (11.3)
		HOMO→LUMO+1 (35.0)			HOMO→LUMO+11 (17.6)
					HOMO→LUMO+12 (24.0)
			2.91	3.54	HOMO-5→LUMO+3 (18.9)
					HOMO-6→LUMO+2 (7.2)
					HOMO-4→LUMO+2 (10.7)
					HOMO-6→LUMO+4 (6.5)
					HOMO-4→LUMO+7 (11.7)
					HOMO→LUMO+26 (17.7)

The authors would like to apologize for any inconvenience caused to the readers by these changes. We will update the paper [1] and the original will remain available on the article webpage.
